# Characteristics of Japanese Duchenne and Becker muscular dystrophy patients in a novel Japanese national registry of muscular dystrophy (Remudy)

**DOI:** 10.1186/1750-1172-8-60

**Published:** 2013-04-19

**Authors:** Harumasa Nakamura, En Kimura, Madoka Mori-Yoshimura, Hirofumi Komaki, Yu Matsuda, Kanako Goto, Yukiko K Hayashi, Ichizo Nishino, Shin‘ichi Takeda, Mitsuru Kawai

**Affiliations:** 1 Institute of Genetic Medicine, Newcastle University, Newcastle upon Tyne, UK; 2 Department of Promoting Clinical Trial and Translational Medicine, Translational Medical Center, National Center of Neurology and Psychiatry, 4-1-1 Ogawa-Higashi, Kodaira, Tokyo, 187-8551, Japan; 3 Department of Neurology, National Center Hospital, National Center of Neurology and Psychiatry, Tokyo, Japan; 4 Department of Child Neurology, National Center Hospital, National Center of Neurology and Psychiatry, Tokyo, Japan; 5 Department of Neuromuscular Research, National Institute of Neurosciences, National Center of Neurology and Psychiatry, Tokyo, Japan; 6 Department of Molecular Therapy, National Institute of Neuroscience, National Center of Neurology and Psychiatry, Tokyo, Japan; 7 Department of Neurology, National Hospital Organization, Higashi-Saitama National Hospital, Saitama, Japan

**Keywords:** Duchenne and Becker muscular dystrophy, Neuromuscular disorder, National registry, TREAT-NMD, Registry of muscular dystrophy (Remudy), Japan

## Abstract

**Background:**

Currently, clinical trials for new therapeutic strategies are being planned for Duchenne and Becker muscular dystrophies (DMD/BMD). However, it is difficult to obtain adequate numbers of patients in clinical trials. As solutions to these problems, patient registries are an important resource worldwide, especially in rare diseases such as DMD/BMD.

**Methods:**

We developed a national registry of Japanese DMD/BMD patients in collaboration with TREAT-NMD. The registry includes male Japanese DMD/BMD patients whose genetic status has been confirmed by genetic analysis. The registry includes patients throughout Japan.

**Results:**

As of February 2012, 583 DMD and 105 BMD patients were registered. Most individuals aged less than 20 years. In terms of genetic mutations of registrants of DMD and BMD, deletion of exons was the most frequent (61.4% and 79.0%) followed by point mutations (24.5% and 14.3%) and duplications (13.6% and 4.8%), respectively. 43.6% of DMD are capable of walking, and 76.2% of BMD registrants are able to walk. 41.1% of DMD registrants in the database were treated using steroids. 29.5% of DMD and 23.8% of BMD registrants were prescribed one cardiac medicine at least. 22% of DMD used ventilator support, and non-invasive support was common. Small numbers of DMD and BMD registrants, only 3.9% and 1.0% of them, have received scoliosis surgery. 57 (9.8%) patients were eligible to clinical trial focused on ‘skipping’ exon 51.

**Conclusions:**

The Remudy has already demonstrated utility in clinical researches and standardization of patients care for DMD/BMD. This new DMD/BMD patient registry facilitates the synchronization of clinical drug development in Japan with that in other countries.

## Introduction

Duchenne muscular dystrophy (DMD) and Becker muscular dystrophy (BMD) are X-linked recessive forms of muscular dystrophy caused by mutations in the dystrophin gene (*DMD*) on chromosome Xp21.2 [1]. The *DMD* gene is the largest gene identified in human and contains 79 exons. Mutations in this gene cause deficiency of normal dystrophin protein [2]. DMD is the most frequently inherited muscular disease, affecting approximately 1 out of 3500 live male new-borns. DMD patients commonly lose their ability to walk before the age of 12 years, coupled with deterioration in respiratory and cardiac functions. DMD is usually fatal in the third decade because of either cardiac or respiratory failure. On the other hand, the course and severity of BMD is more variable [3]. Since the discovery of the *dystrophin gene*, many efforts have been made to develop effective therapeutic strategies for DMD/BMD.

Clinical trials are now being planned and conducted for DMD/BMD [4-8]; however, many challenges exist with regard to both planning and conducting clinical trials for such rare diseases. Because of limited epidemiological data, the total number of patients, natural history of the disease and clinical outcome measures are unclear. In addition, adequate numbers of patients are needed to achieve significant results in clinical trials. As solutions to these problems, patient registries are an important resource especially in case of rare diseases such as DMD/BMD.

In Europe, Translational Research in Europe–Assessment and Treatment of Neuromuscular Diseases (TREAT-NMD) [9], a research network for neuromuscular disorders, developed a global database for DMD and spinal muscular atrophy (SMA) to obtain epidemiological data; examine the total number of patients; determine the natural history of the disease; determine appropriate clinical outcome measures and collect adequate numbers of patients needed to achieve significant results in clinical trials and inform patients of new drug development as soon as possible.

To date, several Japanese DMD/BMD databases have been developed [10-12]; however, these have not been on a broad national scale. For instance, some were on a single-centre basis and others encompassed only a small local area or several hospital sites. Some others were restricted to inpatients only. Despite these early efforts, no national registry has been developed with the purpose of focusing on clinical trials. In 2009, we developed a national registry of Japanese DMD/BMD patients (REgistry of MUscular DYstrophy; Remudy. http://www.remudy.jp/) in collaboration with TREAT-NMD. The purpose of this registry was to effectively recruit eligible patients to new clinical trials and provide timely information to patients about upcoming trials. Registry data also provides more detailed knowledge about the natural history and epidemiology of the disease, as well as information about clinical care. In this paper, we introduce Remudy, the Japanese DMD/BMD registry, and describe the clinical and molecular genetic characteristics of Japanese dystrophinopathy patients.

## Materials and methods

### Institution, organization and leadership

Remudy is supported by a Research Grant (20B-12, 23–4) for Nervous and Mental Disorders from the Ministry of Health, Labour and Welfare. The development and management of the registry is led by the principal investigator of the Japanese muscular dystrophy research group. Steering committee members include scientists, clinicians and representatives of patient organizations. The office of the registry of muscular dystrophy was set up within the National Center of Neurology and Psychiatry (NCNP), Tokyo, Japan. This project includes Japanese DMD/BMD patients and was made possible by collaboration with the Japan Muscular Dystrophy Association.

### Patients

The database includes male Japanese DMD/BMD patients whose genetic status has been confirmed by genetic analysis. The database includes patients throughout Japan. The cost of sequencing analysis of the *DMD* gene is not covered by the system of the public health insurance in Japan. For patients who intend to register but whose genetic status is not confirmed using multiplex ligation-dependent probe amplification (MLPA), Remudy provides free service of sequencing analysis of the *DMD* gene.

### Method of registration and data collection

Information about the registry was provided to interested individuals and their informed consent was obtained. Provision of all data by patients is voluntary and is not shared with any third party without the permission of the committee responsible for disclosing the information. Inclusion in the database confers no obligation for the patient, and they may be removed from the registry immediately on request. It was stated that refusal to participate would not affect the subsequent medical care of the patient. This study was approved by Institutional Review Board of National Center of Neurology and Psychiatry, Japan.

### Structure of the registry form

Data obtained via the registry form included clinical symptoms, results of biochemistry, muscle biopsy and other laboratory analysis and description of the genetic mutation. Epidemiological information provided includes walking capability, cardiac and respiratory functions, creatine kinase levels, history of scoliosis surgery and steroid therapy status. All these data should be confirmed by molecular and clinical curators in Remudy (three active molecular and three active clinical curators). The structure of the Case Report Form and registry items are shown in Table 1. Information was annually updated by registrant’s self report after their physician’s confirmation following reminder from registration office. To decide whether a patient was classified as DMD or BMD, first, attending physician made a diagnosis whether a patient was DMD or BMD by the clinical and molecular information. Then, when our clinical and genetic curators double-checked their classification by reviewing clinical information, and also data from pathological (including dystrophin immuno-staining, if applicable) and genetic analysis.

**Table 1:** The report form for registry to Remudy

**Contact**	**Clinical data**
-Name	-Data
-ID number	-Muscle biopsy
-Hospital	•Examed/not examed
-Date of birth	•Dystrophin immunostain
-Address	-Walking capability
-Phone	•Ambulant /wheelchair
-E-mail	-Use steroid therapy
-Signed up for other registries	-Cardiac function
-Attending any clinical trials	•LVEF(%)
-Registering other database	•Medication
Diagnosis	-Respiratory function
-DMD/BMD/IMD	•FVC
-Proof of the diagnosis	•Mechanical support
•Genetic confirmed	-Scoliosis surgery
•Muscle biopsy	-CK level
•Suspected from family history	-Weight
•Others	Molecular genetic data (certificated report should be attached)
	-Method
	•MLPA/Multiplex PCR/southern blot/RT-PCR/ Direct sequencing of exons
	-Type of mutation
	•Deletion/duplication/others
	•Details of the mutation

DMD, Duchenne muscular dystrophy; BMD, Becker muscular dystrophy; IMD, Intermediate muscular dystrophy; MLPA, Multiplex ligation-dependent probe amplification; LVEF, Left ventricular ejection fraction; FVC, Forced vital capacity; CK, Creatine kinase.

## Results

**Figure 1:**
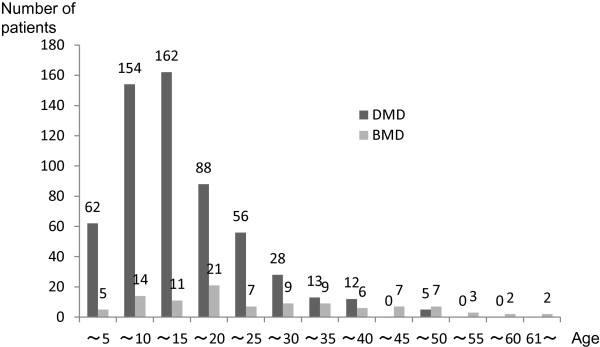
**Ages of registered individuals.** Most registrants are under 20 years of age, but those over 35 years with DMD are also registered.

**Figure 2:**
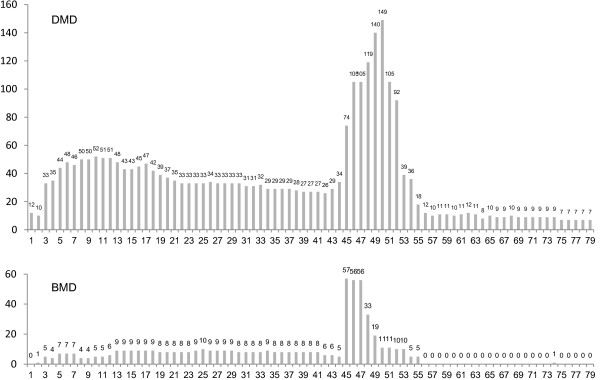
**Frequency of deleted exons observed in registrants with DMD and BMD.** Distribution of exon deletion shows common hot spot regions in exons 45–54 in DMD and BMD.

**Figure 3:**
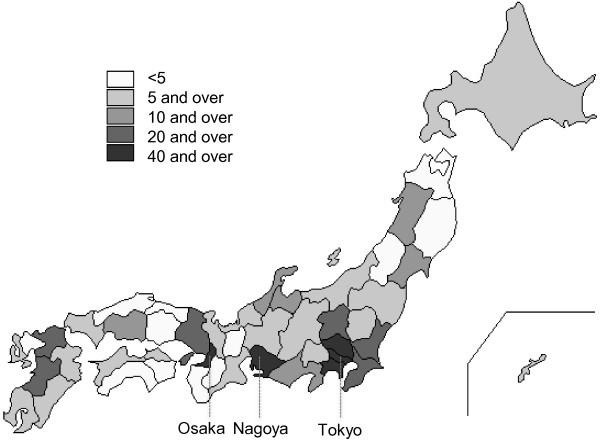
**Prefectural distribution of the registrants.** There are 47 prefectures in Japan. Individuals from all rural areas in Japan were registered but most registrants were concentrated in big cities, namely Tokyo, Osaka and Nagoya.

As of February 2012, 876 Japanese patients across Japan had sought registration, and 583 DMD and 105 BMD patients were registered based on their eligibility as confirmed by clinical and molecular genetic data (Figure 1, Figure 2 and Table 2). Most individuals aged less than 20 years; however, several registered individuals were aged over 35 years. There are five patients between 45 and 49 years old in DMD. The molecular data of four patients are consistent with reading frame rule, and these patients became bedridden with tracheotomy in muscular dystrophy care ward. A molecular data of another patient isn’t consistent with DMD mutation (in-frame mutation, del 10–42), however he lost his walking ability in childhood and had a tracheotomy. His attending doctor clinically diagnosed him as DMD. In terms of genetic mutations of registrants of DMD and BMD, deletion of exons was the most frequent (61.4% and 79.0%) followed by point mutations (24.5% and 14.3%) and duplications (13.6% and 4.8%), respectively. Most registered patients lived in cities, namely Tokyo, Osaka and Nagoya (Figure 3).

**Table 2:** Distribution of mutations in the registrants with DMD and BMD

	**DMD patients**		**BMD patients**	
	**No. of case**	**% of cases**	**No. of case**	**% of cases**
**Distribution of mutation**				
Deletion	358	61.4%	83	79.0%
Duplication	79	13.6%	5	4.8%
Deletion and Duplication	1	0.2%	0	0.0%
Others *	144	24.7%	15	14.3%
No mutation found**	1	0.2%	2	1.9%
	583	100.0%	105	100.0%

* Others include nonsense mutations, small insertion/deletion mutations, deep intronic mutations, and splice site mutations.

** Their diagnosis was confirmed based on their pathological findings in muscle biopsy including a negative immunohistochemical staining against dystrophin.

Tables 3 describe the clinical characteristics of Japanese DMD/BMD patients included in the registry. The information collected was similar to that for the core items required for the TREAT-NMD global registry, and thus, we will be able to compare Japanese data with those contained in the global registry.

**Table 3:** Clinical manifestations, medications and intervention characteristics in the registrants with DMD and BMD

	**DMD patients**		**BMD patients**	
	**No. of case**	**% of cases**	**No. of case**	**% of cases**
**Walking capability**				
Normal walking	254	43.6%	80	76.2%
Not able to walk, and sit without support	184	31.6%	19	18.1%
Not able to sit without support	145	24.9%	6	5.7%
	583	100.0%	105	100.0%
**Cardiac function**				
Normal	392	67.2%	76	72.4%
Dysfunction	180	30.9%	28	26.7%
Not performed	11	1.9%	1	1.0%
	583	100.0%	105	100.0%
**Respiratory function**				
Normal	65	11.1%	45	42.9%
Dysfunction	202	34.6%	18	17.1%
Not performed	316	54.2%	42	40.0%
	583	100.0%	105	100.0%
**Steroid use**				
Current	171	29.3%	6	5.7%
Used to	69	11.8%	6	5.7%
Never	343	58.8%	93	88.6%
	583	100.0%	105	100.0%
**Cardiac medication**				
Prescribed	172	29.5%	25	23.8%
Not prescribed	411	70.5%	80	76.2%
	583	100.0%	105	100.0%
**Drug**				
β-blocker	94	54.7%	16	64.0%
ACE-inhibitor	140	81.4%	19	76.0%
ARB	12	7.0%	5	20.0%
Diuretics	43	25.0%	7	28.0%
Other	29	16.9%	7	28.0%
	172^*^1	100%	25^*^1	100%
**Mechanical ventilation**				
No	455	78.0%	103	98.1%
Yes	128	22.0%	2	1.9%
	583	100.0%	105	100.0%
Invasive	20	15.6%	2	100.0%
Non-invasive	108	84.4%	0	0.0%
	128^*2^	100.0%	2^*2^	100.0%
Temporal	71	55.5%	0	0.0%
Continuous	57	44.5%	2	100.0%
	128*2	100.0%	2*2	100.0%
**Scoliosis surgery**				
Yes	23	3.9%	1	1.0%
No	560	96.1%	103	98.1%
Not described	0	0.0%	1	1.0%
	583	100.0%	105	100.0%

*1; The number of registrants who were prescribed with cardiovascular medicines.

*2; The number of registrants using ventilator support.

Among the DMD registrants, 43.6% of them are capable of walking. On the other hand, 76.2% of BMD registrants are able to walk. In terms of cardiac and respiratory function, 67.2% of DMD and 72.4% of BMD have normal cardiac function and respiratory functional examination was not performed among half of DMD registrants. It was suggested that many registrants were too young to be tested when they were registered. 41.1% of DMD registrants in the database were treated using steroids. 29.5% of DMD and 23.8% of BMD registrants were prescribed one cardiac medicine at least, and ACE–inhibitor was the most common medicine for cardiac failure. One fifth of DMD used ventilator support, and among the registrants with mechanical ventilation, non-invasive support was common. Small numbers of DMD and BMD registrants, only 3.9% and 1.0% of them, have received scoliosis surgery. The Remudy registry has already demonstrated utility in clinical trials for DMD/BMD. A clinical trial focused on ‘skipping’ exon 51 using antisense oligonucleotides has been recently conducted in Japan. From the Remudy registry data, 57 (9.8%) patients have mutations theoretically addressable by skipping of exon 51 (Table 4).

**Table 4:** Applicable individuals for exon 51 skipping clinical trial

**Deleted exons**	**No. of individuals**	**% in registrants**
45-50	17	2.9%
47-50	0	0.0%
48-50	9	1.5%
49-50	18	3.1%
50	6	1.0%
52	7	1.2%
52-63	0	0.0%
total	57	9.8%

## Discussion

The DMD/BMD registry run by Remudy is the one of its kind in Japan specifically targeting neuromuscular diseases. It is the first national registry in Japan to include both clinical and molecular genetic data, and its utility in promoting clinical trials has been demonstrated. Prior to the development of Remudy, some DMD/BMD databases existed in Japan [10-12]; however, they were limited in their coverage and utility. This is the first Japanese DMD/BMD database aimed at facilitating clinical trials and the first to coordinate with a global database.

Since 2011, a worldwide phase III, randomized, double-blind, placebo-controlled clinical study is being conducted for patients with DMD who have a *DMD* gene mutation amenable to an exon 51 skip. Partially because of the development of the Remudy database, this trial now includes Japanese sites and is thus the first global clinical trial to become available to Japanese DMD patients. Up until now, clinical trials for DMD under new International Conference on Harmonisation of Technical Requirements for Registration of Pharmaceuticals for Human Use–Good Clinical Practice (ICH-GCP) have not been conducted in Japan. Registry information concerning eligible Japanese individuals was sought by TREAT-NMD and pharmaceutical companies and has been provided with permission. This example demonstrates the usefulness of the registry for facilitating patient access to clinical trials, as well as enhancing recruitment for such trials.

Another novel aspect is patient participation in the registration process. Previously, database information was supplied by clinicians. In contrast, the Remudy system enables patients themselves to provide their information to the registry in collaboration with their clinicians. The registry consists of valuable clinical and genetic data, which yields valuable epidemiological information including walking capability, cardiac and respiratory functions, creatine kinase levels, history of scoliosis surgery and steroid therapy status, all of which are needed to plan clinical trials and determine the eligibility of individuals for such trials. This has resulted in Remudy becoming one of the largest and most reliable rare disease registries in Japan.

Remudy also reveals the structure of mutations in Japanese DMD/BMD patients. The distribution of mutations and the frequency of individual exon deletion found in our study are consistent with previous reports. In DMD, distribution, duplication and point mutations comprise 61.4%, 13.6% and 24.5%, respectively. Distribution of exon deletions reveals significant region in exons 42–54 (Figure 2). This is similar to the findings of other databases [13] and those of a Japanese study in a single referral centre [11].

Because of improvements in respiratory and cardiac complications, life expectancy of Japanese DMD patients has been prolonged [14]. Our findings support this, with the Remudy registry including several individuals over 40 years of age (though most were aged <20 years). 41.1% of DMD patients in the database were treated using steroids. Importantly, this ratio may be relatively low compared to that in western countries, considering that the family guide for the diagnosis and management of DMD became available to download in Japanese in 2011 [15]. Given that this treatment is recommended in the guide, the number of individuals being treated with steroids for DMD may increase in future.

Remudy is intended to be used as a public service for the benefit of patients living with neuromuscular diseases. Through participation in the database, patients may be identified as candidates for upcoming clinical trials. In addition, Remudy has provided additional useful resources for patients through a website and newsletter. The information provided via these methods includes the development of new therapeutic compounds, information regarding care standards (known as the Family Guide for the Diagnosis and Management of Duchenne Muscular Dystrophy [15]) and other relevant medical information. In addition, Remudy has facilitated the genetic diagnosis and detailed sequencing analysis of individuals, thereby promoting genetic counseling in family and also providing important information required to determine eligibility for clinical trials. As a result, more dystrophinopathy patients have become aware of the necessity of confirming their diagnosis via analysis of the *DMD* gene.

The development of Remudy is part of a global trend toward database development for neuromuscular disorders. TREAT-NMD is an excellent network in the field of neuromuscular disorders and has been leading international registry collaboration for many diseases including DMD, spinal muscular atrophy, myotonic dystrophy type 1 and others. Close collaboration with TREAT-NMD has allowed Japan to enhance the management and care of Japanese DMD and BMD patients and facilitated research in neuromuscular disorders. One of the most important outcomes of this collaboration is the development of the Japanese national registry for DMD described in this paper. By collecting common information, the registry data can easily be compared between Japan and other countries. TREAT-NMD has helped to support these efforts by providing an infrastructure that continues to accelerate research, therapy development, and trial readiness in addition to increasing collaboration and improving patient care. Japanese patients, families, physicians and anyone affected by a neuromuscular disorder play a key role in the worldwide neuromuscular community by participating in this collaboration with TREAT-NMD, which is Remudy.

The TREAT-NMD global registry has been focused on European countries and the United States; however, it is currently expanding to other countries. Rare disease registries are emerging in Asian countries [16] and Japanese experiences in collaboration with TREAT-NMD and registry development should be able to provide more information about these activities to Asian countries. A global registry for patients with myotonic dystrophy type 1 will be launched in the near future [17]. Several other registries for neuromuscular diseases have been developed worldwide, such as the international congenital muscular dystrophy registry (CMDIR) run by the US patient organization ‘Cure CMD’. Remudy has been developing Japanese registries for other neuromuscular diseases including glucosamine (UDP-N-acetyl)–2-epimerase/N-acetylmannosamine kinase (GNE) myopathy and myotonic dystrophy type 1 (DM1).

We have thus demonstrated how this registry can enhance the readiness for clinical trials in Japan, and how this unique form of infrastructure can be used to accelerate international efforts in fighting orphan diseases.

## Conclusions

The Remudy registry has already demonstrated utility in clinical trials and standardization of patients care for DMD/BMD. This new DMD/BMD patient registry will facilitate the synchronization of clinical drug development in Japan with that in other countries.

## Competing interests

The authors declare that they have no competing interests.

## Authors’ contributions

HN and EK participated in planning this study, analysis and interpretation, generation of the tables and figures, in writing of the manuscript. MY and HK participated in curating clinical data and data collection. YM, KG, and YH participating in genetic analysis, curating the molecular data and data collection. IN, ST and MK supervised in planning this study. All authors read and approved of the final manuscript.
